# Interface Engineering of CoFe-LDH Modified Ti: α-Fe_2_O_3_ Photoanode for Enhanced Photoelectrochemical Water Oxidation

**DOI:** 10.3390/nano13182579

**Published:** 2023-09-18

**Authors:** Yue Chang, Minmin Han, Yehui Ding, Huiyun Wei, Dawei Zhang, Hong Luo, Xiaogang Li, Xiongbo Yan

**Affiliations:** 1 Institute of Advanced Materials and Technology, University of Science and Technology Beijing, Beijing 100083, China; 2 National Materials Corrosion and Protection Data Center, University of Science and Technology Beijing, Beijing 100083, China; 3 BRI Southeast Asia Network for Corrosion and Protection (MOE), Shunde Innovation School, University of Science and Technology Beijing, Foshan 528399, China; 4 National Engineering Research Center for Intelligent Electrical Vehicle Power System, College of Mechanical and Electrical Engineering, Qingdao University, Qingdao 266071, China; 5 International Center for Materials Nanoarchitectonics (WPI-MANA), National Institute for Materials Science (NIMS), 1-1 Namiki, Tsukuba 305-0044, Ibaraki, Japan; 6 School of Mathematics and Physics, University of Science and Technology Beijing, Beijing 100083, China; 7 Beijing Advanced Innovation Center Materials Genome Engineering, University of Science and Technology Beijing, Beijing 100083, China

**Keywords:** CoFe-LDH, titanium doping, hematite, interface coupling enhancement, photoelectrochemical water oxidation

## Abstract

Effectively regulating and promoting the charge separation and transfer of photoanodes is a key and challenging aspect of photoelectrochemical (PEC) water oxidation. Herein, a Ti-doped hematite photoanode with a CoFe-LDH cocatalyst loaded on the surface was prepared through a series of processes, including hydrothermal treatment, annealing and electrodeposition. The prepared CoFe-LDH/Ti:α-Fe_2_O_3_ photoanode exhibited an outstanding photocurrent density of 3.06 mA/cm^2^ at 1.23 V_RHE_, which is five times higher than that of α-Fe_2_O_3_ alone. CoFe-LDH modification and Ti doping on hematite can boost the surface charge transfer efficiency, which is mainly attributed to the interface interaction between CoFe-LDH and Ti:α-Fe_2_O_3_. Furthermore, we investigated the role of Ti doping in enhancing the PEC performance of CoFe-LDH/Ti:α-Fe_2_O_3_. A series of characterizations and theoretical calculations revealed that, in addition to improving the electronic conductivity of the bulk material, Ti doping also further enhances the interface coupling of CoFe-LDH/α-Fe_2_O_3_ and finely regulates the interfacial electronic structure. These changes promote the rapid extraction of holes from hematite and facilitate charge separation and transfer. The informative findings presented in this work provide valuable insights for the design and construction of hematite photoanodes, offering guidance for achieving excellent performance in photoelectrochemical (PEC) water oxidation.

## 1. Introduction

Photoelectrochemical (PEC) water splitting has attracted considerable attention owing to the urgent requirement for sustainable and clean hydrogen energy. This technology holds the potential to effectively tackle the economic and environmental challenges associated with fossil fuels [1,2]. The process of PEC water splitting, which separates water into hydrogen and oxygen, involves a complex multi-electron reaction, driven by the generation of electron–hole pairs within a semiconductor catalyst under light irradiation. In PEC water splitting, the oxygen evolution reaction (OER) requires four electrons to complete, making it more challenging than the two-electrons hydrogen evolution reaction (HER) [3,4]. In this case, the development of efficient photoanode materials has become one of pivotal problems for PEC water splitting. Among various photoanode materials, hematite stands out due to its narrow band gap (~2.1 eV), its high theoretical solar-to-hydrogen efficiency (15.3%), its abundance in the Earth’s crust and its good chemical stability [5,6]. However, the PEC performance of hematite can be limited, which is attributed to the high rate of recombination of photoinduced electron–hole pairs, and the low efficiency of hole injection. The limitations result in practical efficiencies far below the theoretical values [7,8]. 

The surface modification of hematite with oxygen evolution cocatalysts (OECs) has been demonstrated as an effective approach to address the issues mentioned above [9,10,11]. Layered double hydroxides (LDHs), which contain edge-sharing octahedral MO_6_ units, are ideal candidates due to their unique layered structure and compositional versatility. Previous work has reported on introduced LDHs, such as NiFe-LDHs [12], NiCe-LDHs [13], ZnCo-LDHs [14] and CoAl-LDHs [15], onto hematite photoanodes to enhance PEC performance. Among the LDHs, CoFe-LDHs have attracted research interest for (photo)electrocatalytic water oxidation due to the synergistic effect of Fe and Co, which can enhance OER activity [16,17,18]. Xu et al. [19] reported on engineering interfaces to steer hole dynamics in BiVO_4_ photoanodes for solar water oxidation. CoFe-LDHs were decorated on the surface of a BiVO_4_/Co_3_O_4_ photoanode using cathodic electrodeposition, demonstrating further enhancement in PEC performance. However, an undesirable interface between the semiconductor and LDHs cocatalyst can have a detrimental effect on PEC water oxidation. 

Elemental doping combined with surface modification represents one of the most effective strategies for addressing the low activity of hematite in PEC water oxidation [20,21]. Metal ion dopants, such as Ti^4+^ acting as donor impurities, have been widely reported for their role in enhanced PEC performance of hematite photoanodes [22,23]. Ti^4+^ doping can improve the electronic conductivity of hematite by regulating its electronic structure. For surface modified photoanodes, such as OEC decorated hematite, the regulated electronic structure and increased carrier density of hematite inevitably result in the change in interface states between the OECs and hematite [24,25]. Consequently, during PEC water oxidation, the charge separation and transfer on the surface and interface are affected. Therefore, it is expected that Ti doping may improve surface or interface charge transfer and enhance favorable interactions at the interface, rather than just solely focusing on changes in bulk conductivity resulting from donor doping. 

Inspired by the above discussion, we successfully fabricated a CoFe-LDH/Ti:α-Fe_2_O_3_ photoanode via the hydrothermal treatment–annealing–electrodeposition method. Ti doping played an important role for the CoFe-LDH/α-Fe_2_O_3_ photoanode for enhancing the PEC performance of water oxidation. We found that Ti doping not only improves the charge separation of bulk hematite, but also enhances the surface charge transfer and charge injection into the electrolyte by enhancing the interface coupling effect and regulating the interfacial electronic structure between CoFe-LDH and α-Fe_2_O_3_. This work contributes to a deeper understanding of the mechanisms behind the enhancement of photoanodes for photoelectrochemical (PEC) water oxidation, providing new insights on improving surface and interface charge separation and transfer.

## 2. Experimental Details

### 2.1. Synthesis of Photoanodes

According to previous reports [22,26], the hematite materials with or without Ti doping were prepared on the conductive surface of fluorine-doped tin oxide substrates (14 Ω/cm^2^, Wuhan Lattice Solar Technology, Wuhan, China) via a hydrothermal–annealing method. More specifically, firstly, the precursor solution was prepared through dissolving 0.15 M FeCl_3_·6H_2_O (AR, Aladdin, Shanghai, China) and 1 M NaNO_3_ (AR, Sinopharm Chemical Reagent, Shanghai, China). For Ti-doped hematite materials, 0.11 M titanium tetrachloride (AR, Aladdin, Shanghai, China) in ethanol solution with a volume of 16 μL was added to the precursor solution, in which the Ti/Fe content ratio in feed was consistent with 0.3 wt%. After that, five pieces of cleaned fluorine-doped tin oxide glass were placed into a Teflon-lined autoclave, in which the fluorine-doped tin oxide conductive was face down against the wall of the autoclave. The prepared precursor solution was added into the autoclave and immersed the fluorine-doped tin oxide glass. This autoclave was then heated to 100 °C for 4 h. The obtained yellow materials attached to the surface of the fluorine-doped tin oxide glass were β-FeOOH or Ti-doped β-FeOOH. Finally, the obtained samples were washed with deionized water, and annealed in air at 550 °C for 2 h in a muffle furnace and at 700 °C for 20 min in a muffle furnace. The as-prepared Ti-doped α-Fe_2_O_3_ films were designated as TFO.

According to previous reports [19,27], CoFe-LDH materials were prepared on the surface of the hematite substrates, with or without Ti doping, through a simple electrodeposition method. In a typical procedure, we prepare a mixture of 0.006 M Co(NO_3_)_2_ (AR, Energy Chemical, Shanghai, China) and 0.006 M Fe(NO_3_)_3_ (AR, Aladdin, Shanghai, China) in aqueous solutions as the electrodeposited electrolyte. A three-electrode configuration was used, where the substrate served as the working electrode, a platinum plate as the counter electrode, and an Ag/AgCl electrode as the reference electrode. The electrodeposition of CoFe-LDH was conducted under a constant voltage of −0.8 V_Ag/AgCl_ with a deposition time of 80 s. Subsequently, the working electrodes were thoroughly washed with copious amounts of water. The obtained samples with different substrates were designated as CoFe-LDH/α-Fe_2_O_3_ and CoFe-LDH/TFO, respectively.

### 2.2. Characterization

The morphology was observed through a ZEISS GeminiSEM 300 field emission scanning electron microscope (FE-SEM, ZEISS, Oberkochen, Germany) and an FEI Tecnai G2 F20 transmission electron microscope (TEM, FEI, Oberkochen, Germany). The X-ray diffraction (XRD) patterns for all the samples were analyzed using a Rigaku SmartLab X-ray diffractometer (Cu Kα radiation, Rigaku, Tokyo, Japan) with a scan range of 2θ from 10 to 80° and a scan rate of 6°/min. The diffuse UV–Vis absorption spectra were recorded using a Shimadzu spectrophotometer (UV-2700, Shimadzu, Kyoto, Japan) equipped with an integral sphere in the range of 300–800 nm. The XPS spectra of the samples were measured by a PHI Quantera II spectrometer (ULVAC-PHI, Kanagawa, Japan), using Al Kα X-ray as the excitation source. All the spectra were calibrated using the C1s binding energy at 284.8 eV as the reference.

### 2.3. Photoelectrochemical Measurements

The photoelectrochemical (PEC) experiments were performed on a CHI660E electrochemical workstation in a typical three-electrode configuration with a 1 M NaOH aqueous solution serving as the electrolyte. The prepared photoanodes were employed as the working electrode. A platinum plate (1 × 1 cm^2^) and a Ag/AgCl electrode were employed as the counter electrode and the reference electrode, respectively. The exposed area of the photoanode immersed in the aqueous electrolyte was controlled to be 0.2826 cm^2^. A 300 W Xe lamp equipped with an AM 1.5 filter was used to simulate solar light irradiation, and the light intensity was adjusted to 100 mW/cm^2^. All the measured potentials (vs. *Ag/AgCl*) were converted to the *RHE* scale using the following Equation (1):

(1) $$E_{RHE}=E_{Ag/AgCl}+0.059pH+E_{Ag/AgCl}^{0}$$

where $E_{RHE}$ and $E_{Ag/AgCl}$ refer to the converted potential vs. the RHE and the measured potential vs. Ag/AgCl, respectively. $E_{Ag/AgCl}^{0}=0.197\;V$ at 25 °C. The photocurrent-potential curves were generated through linear sweep voltammetry (LSV) measurements within a voltage range of 0.5 V to 1.5 V vs. the RHE at a scanning rate of 10 mV/s. The transient photocurrent densities were recorded in cycles of 20 s at a potential of 1.23 V vs. the RHE. The applied bias photon-to-current efficiency (*ABPE*) was calculated using the following Equation (2):

(2) $$ABPE(\%)=\frac{J\times (1.23-V_{b})}{P_{total}}$$

where J represents the photocurrent density (mA/cm^2^), $V_{b}$ is the applied bias vs. the RHE, and $P_{total}$ refers to the total light intensity of AM 1.5 G (100 mW/cm^2^). The IPCE measurements for all the photoanodes were conducted using a monochromator (Omno151, NBET, Beijing, China). The IPCE spectra were measured at 1.23 V vs. the RHE, with 10 nm steps in the wavelength range of 350 nm~600 nm. The incident photon-to-current conversion efficiency (*IPCE*) is calculated using the following Equation (3):

(3) $$IPCE=\frac{1240\times I}{\lambda \times P_{light}}$$

where λ represents the wavelength of the incident light, and I and $P_{light}$ are the measured photocurrent density and irradiance at some specific wavelengths. The water oxidation photocurrent density can be calculated using the following Equation (4):

(4) $$J_{PEC}=J_{abs}\times \eta_{sep}\times \eta_{inj}$$

where $J_{abs}$ represents the photocurrent density from the photon completely absorbed into the current. As a hole scavenger, Na_2_SO_3_ can effectively trap the holes that arrive at the surface, and there is no influence on the charge separation on the electrode ($\eta_{inj}$ assumed to be 100%). Therefore, the charge separation efficiency of the bulk ($\eta_{sep}$) and the charge transfer efficiency of the surface ($\eta_{inj}$) can be given by the following Equations (5) and (6):

(5) $$\eta_{sep}=\frac{J^{{Na}_{2}{SO}_{3}}}{J_{abs}}$$

(6) $$\eta_{trans}=\frac{J^{H_{2}O}}{J^{{Na}_{2}{SO}_{3}}}$$

where $J^{H_{2}O}$ and $J^{{Na}_{2}{SO}_{3}}$ represent the photocurrent densities measured in 1 M NaOH electrolyte without and with 1 M Na_2_SO_3_, respectively. The EIS measurements were conducted with 1.23 V_RHE_ potential over the frequency range of 0.05 Hz to 1 MHz under simulated solar irradiation. Mott–Schottky data were collected in the dark at a scan rate of 50 mV/s with a frequency of 1 kHz in the potential range from −0.5 to 0.5 V vs. Ag/AgCl.

## 3. Results and Discussion

### 3.1. C and Characterization of the As-Synthesized Photoanode Materials

**Figure 1:**
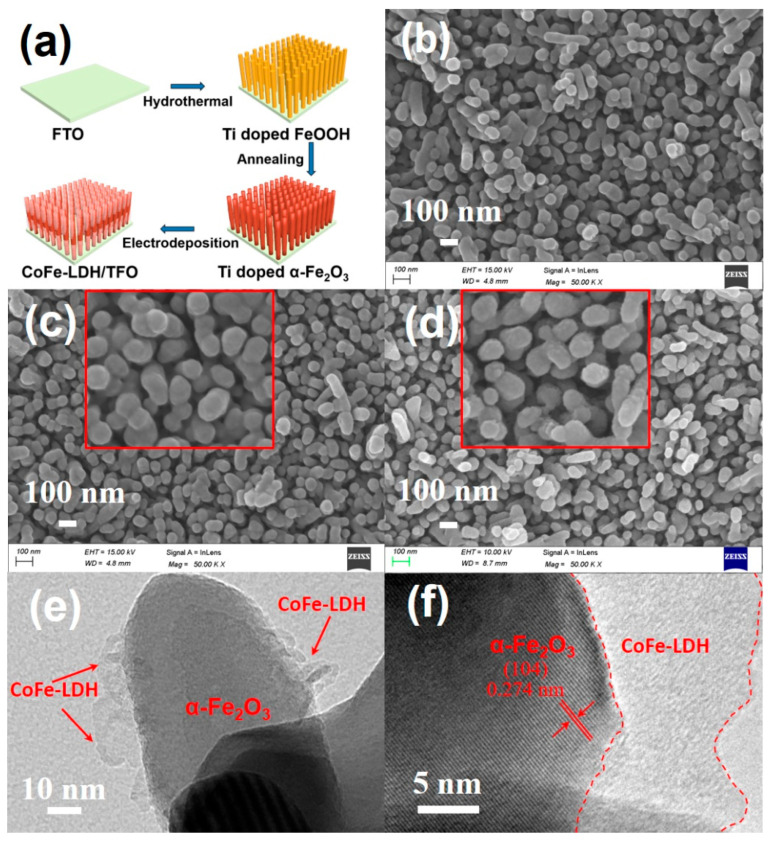
(**a**) The synthesis procedure of CoFe-LDH/Ti:α-Fe_2_O_3_ photoanode; SEM images of (**b**) α-Fe_2_O_3_, (**c**) TFO and (**d**) CoFe-LDH/TFO; (**e**) TEM image and (**f**) HRTEM image of CoFe-LDH/TFO.

The fabrication process of the CoFe-LDH/Ti:α-Fe_2_O_3_ film is illustrated in Figure 1a. Firstly, yellow Ti-doped β-FeOOH nanorods were grown on a conductive glass substrate using the hydrothermal method and, subsequently, transformed into red Ti-doped α-Fe_2_O_3_ (TFO) through annealing. Finally, the CoFe-LDH was decorated on the surface of the TFO via a common cathodic electrodeposition method for LDH materials [19,27]. Figure 1 and Figure S1 (Supplementary Materials) display the SEM and TEM images of the α-Fe_2_O_3_, TFO and CoFe-LDH/TFO samples. The vertically aligned nanorods of α-Fe_2_O_3_ can be observed in Figure 1b–d, with the average diameters ranging from 70 to 100 nm and a thickness of approximately 500 to 600 nm (Figure S1a). This indicates that the nanorod-like structure is still well preserved (Figure 1c), although the surface of the CoFe-LDH/TFO appears slightly rough (Figure 1d). The TEM and HRTEM images in Figure 1e,f further confirm the nanosheet morphology of CoFe-LDH with a thickness of 2 to 10 nm. Additionally, the HRTEM image clearly reveals a lattice spacing of 0.274 nm, corresponding well to the (104) plane of α-Fe_2_O_3_. In another region, no lattice fringes are visible, indicating the amorphous state of CoFe-LDH. The EDS elemental mapping images for a selected region are shown in Figure S1b. These results demonstrated the even distribution of the Fe, O, Ti and Co elements, indicating the successful doping of the Ti element and decoration of the CoFe-LDH nanosheets on hematite. 

The XRD patterns of the α-Fe_2_O_3_, TFO and CoFe-LDH/TFO films in Figure S1c exhibit similar characteristic peaks. These diffraction peaks can all be indexed to α-Fe_2_O_3_ (JCPDS No. 86-0550) and SnO_2_ derived from the fluorine-doped tin oxide substrate (JCPDS No. 41-1445). There are no obvious changes in the diffraction peaks after Ti doping and CoFe-LDH deposition. This is due to the low concentration of Ti dopants and the amorphous structure of CoFe-LDH, which is consistent with the HRTEM results. The DRS UV–Vis spectra of the α-Fe_2_O_3_, TFO, CoFe-LDH/α-Fe_2_O_3_ and CoFe-LDH/TFO photoanodes are presented in Figure S1d. The absorption edge of α-Fe_2_O_3_ is located at approximately 583 nm. It can be observed that the introduction of CoFe-LDH does not significantly alter the absorption spectra of CoFe-LDH/α-Fe_2_O_3_ and CoFe-LDH/TFO compared with α-Fe_2_O_3_ and TFO, respectively. This indicates the good optical transparency of CoFe-LDH. However, Ti doping leads to a slight redshift in the absorption edges for the TFO and CoFe-LDH/TFO samples compared with α-Fe_2_O_3_ and CoFe-LDH/α-Fe_2_O_3_, respectively. The band gap energies of the above samples were calculated based on the (α*hv*)^2^-*hv* curves [28] (Figure S2b (inset)). The *E*_g_ values of α-Fe_2_O_3_ and CoFe-LDH/α-Fe_2_O_3_ are approximately 2.08 eV, while those of TFO and CoFe-LDH/TFO are around 2.13 eV. This suggests that the Ti elements were successfully doped into the bulk of α-Fe_2_O_3_, resulting in a slight enhancement of the visible light absorption.

**Figure 2:**
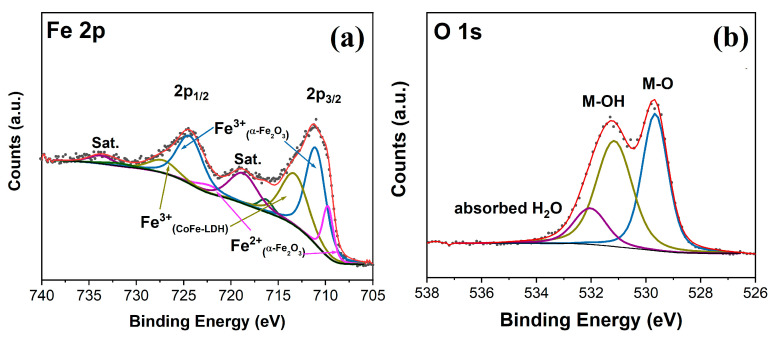

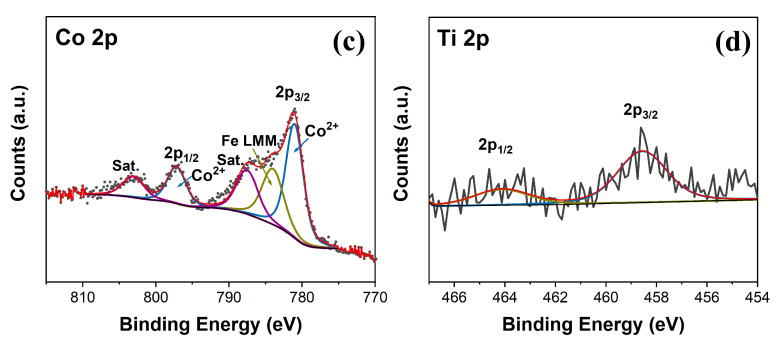
High-resolution XPS spectra of (**a**) Fe 2p; (**b**) O 1s; (**c**) Co 2p and (**d**) Ti 2p in the CoFe-LDH/TFO samples.

To further confirm the electrodeposition of CoFe-LDH on the surface of the TFO, the XPS analysis of the CoFe-LDH/TFO samples was conducted to determine the surface elemental composition and chemical state. The high-resolution XPS spectra of Fe 2p, O1s, Co 2p and Ti 2p are presented in Figure 2a–d. As shown in Figure 2a, in the Fe 2p XPS spectrum, the binding energies of 711.0 eV and 724.5 eV correspond to the peaks of Fe 2p_3/2_ and Fe 2p_1/2_, respectively. Followed by fitting deconvolution, the two peaks at 713.3 eV and 727.2 eV are assigned to the Fe^3+^ in CoFe-LDH, the two peaks of lower binding energy centered at 711.1 eV and 724.3 eV represent the Fe^3+^ in α-Fe_2_O_3_, and the other two peaks at 709.7 eV and 722.4 eV are assigned to the Fe^2+^ of α-Fe_2_O_3_ [29,30]. Additionally, the observable Fe^3+^ satellite peaks at 718.7 eV and 733.8 eV are consistent with the presence of Fe^3+^ in α-Fe_2_O_3_ and CoFe-LDH [31,32]. The O1s peaks of the CoFe-LDH/TFO samples can be deconvoluted into three peaks, as seen in Figure 2b. The binding energies centered at 529.8 eV, 531.3 eV and 532.2 eV can be assigned to the lattice oxygen species in hematite, the hydroxyl groups of CoFe-LDH and the absorbed H_2_O, respectively [33]. In Figure 2c, the Co 2p XPS spectrum shows two major peaks at binding energies of Co 2p_3/2_ and Co 2p_1/2_, accompanying two satellite peaks located at 787.5 eV and 803.1 eV. After further fitting of the Co 2p_3/2_ and Co 2p_1/2_, the binding energies at 781.0 eV and 797.2 eV can be attributed to the Co^2+^ in CoFe-LDH [34]. Moreover, the Ti 2p XPS spectrum of the CoFe-LDH/TFO samples is presented in Figure 2d. The peaks at 458.2eV and 464.1eV correspond to the Ti^4+^ 2p_3/2_ and Ti^4+^ 2p_1/2_ peaks, respectively, indicating the presence of Ti^4+^ in the TFO [23]. The observation of the Ti 2p XPS spectra confirms the successful doping of Ti, which is consistent with the results of the DRS UV–Vis analysis.

### 3.2. The PEC Performance of Water Oxidation for Photoanodes

**Figure 3:**
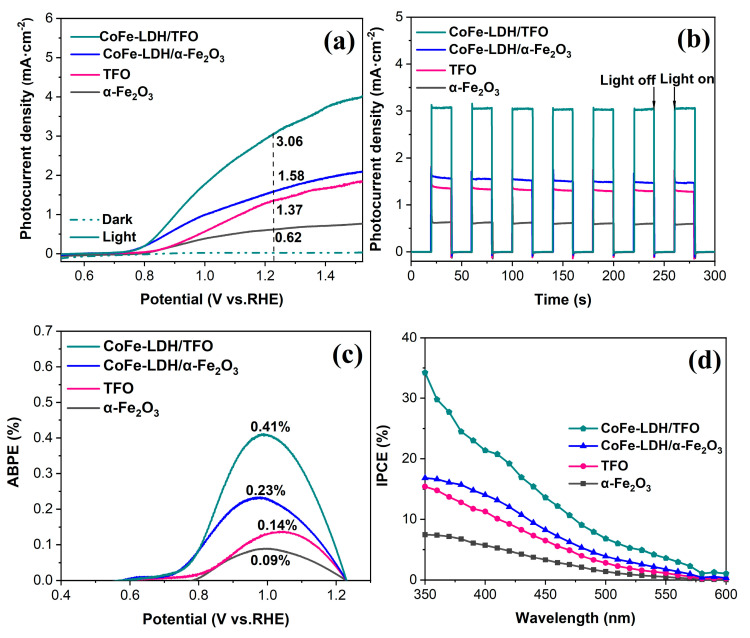
(**a**) LSV curves, (**b**) photocurrent-time curves at 1.23 V_RHE_, (**c**) ABPE curves and (**d**) IPCE curves at 1.23 V_RHE_ of α-Fe_2_O_3_, TFO, CoFe-LDH/α-Fe_2_O_3_ and CoFe-LDH/TFO photoanodes.

The PEC performance of the as-prepared α-Fe_2_O_3_, TFO, CoFe-LDH/α-Fe_2_O_3_ and CoFe-LDH/TFO photoanodes was investigated through LSV curves under light irradiation. As exhibited in Figure 3a, the α-Fe_2_O_3_ photoanode presents a low photocurrent density of 0.62 mA/cm^2^ at 1.23 V_RHE_. The TFO photoanode, on the other hand, displays a higher photocurrent density (1.37 mA/cm^2^ at 1.23 V_RHE_), attributed to the Ti dopant increasing the donor density and enhancing the electrical conductivity of the α-Fe_2_O_3_ film [22,23]. By further electrodepositing CoFe-LDH on the surface of α-Fe_2_O_3_ and TFO, the photocurrent densities are both increased, reaching 3.06 mA/cm^2^ at 1.23 V_RHE_. Notably, the current of CoFe-LDH/TFO in the dark is negligible, indicating that the improvement in the photocurrent arises from the photoelectric conversion process. An apparent cathodic shift in the onset potential is observed for CoFe-LDH/TFO and CoFe-LDH/α-Fe_2_O_3_, shifting from 0.81 V_RHE_ of α-Fe_2_O_3_ and 0.80 V_RHE_ of TFO to 0.72 V_RHE_ of CoFe-LDH/α-Fe_2_O_3_ and 0.74 V_RHE_ of CoFe-LDH/TFO, respectively. Moreover, the steady-state photocurrents were determined by the I-t curve at 1.23 V_RHE_ (Figure 3b and Figure S2), and the results are in good agreement with the LSV results. The photocurrent density of the CoFe-LDH/TFO photoanodes remains constant over 3600 s of light irradiation. Figure 3c illustrates the applied bias photo-to-current efficiency (ABPE) values of all the photoanodes calculated from the LSV curves. The ABPE values of CoFe-LDH/TFO reach up to 0.41% at 1.00 V_RHE_, significantly exceeding those of TFO (0.14% at 1.03 V_RHE_), α-Fe_2_O_3_ (0.09% at 1.01 V_RHE_) and CoFe-LDH/α-Fe_2_O_3_ (0.23% at 0.97 V_RHE_). The incident photon-to-current conversion efficiency (IPCE) was also obtained in the wavelength range of 350–600 nm. According to Figure 3d, the CoFe-LDH/TFO photoanodes exhibit the highest IPCE values (34.2% at 350 nm), which are four times higher than those of α-Fe_2_O_3_ (7.6% at 350 nm). These results indicate that the enhancement of the PEC performance in CoFe-LDH/TFO can be mainly attributed to the synergistic effects of the Ti dopant and CoFe-LDH nanosheets.

### 3.3. The Effect of Ti Dopants and CoFe-LDH Deposition on the Charge Transfer Process of the Photoanodes

**Figure 4:**
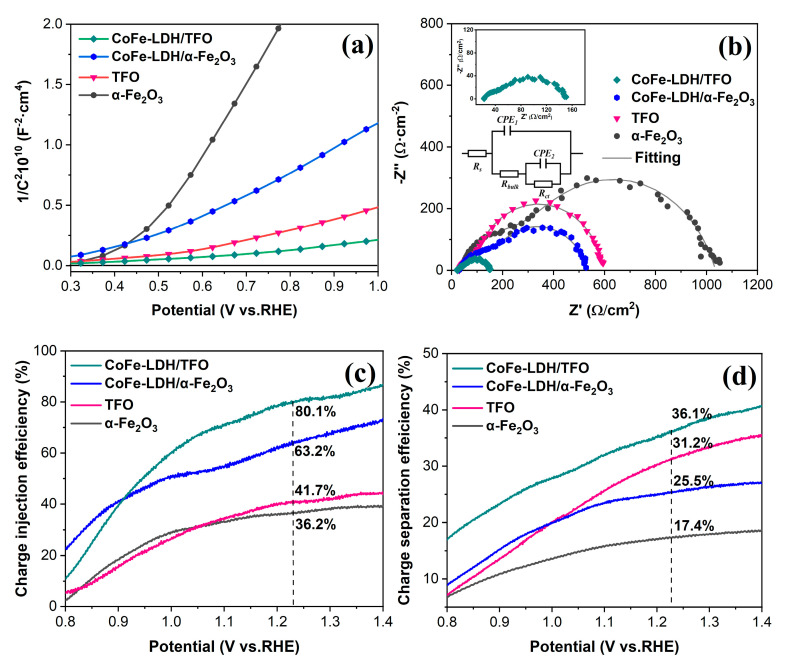
(**a**) Mott–Schottky plots, (**b**) EIS at 1.23 V_RHE_ under light irradiation (R_s_, R_bulk_, R_ct_ and CPE refer to the series resistance, the charge trapping resistance in bulk with high frequency, the charge transfer resistance across the interface of the photoanode and electrolyte with low frequency and the constant phase element, respectively), (**c**) the charge injection efficiency and (**d**) charge separation efficiency of α-Fe_2_O_3_, TFO, CoFe-LDH/α-Fe_2_O_3_ and CoFe-LDH/TFO photoanodes.

To understand the catalytic effect of Ti doping and CoFe-LDH deposition, we investigated the charge transfer and separation behaviors through a series of electrochemical measurements. Mott–Schottky plots were used to determine the carrier densities (N_d_) and flat-band potentials of the α-Fe_2_O_3_, TFO, CoFe-LDH/α-Fe_2_O_3_ and CoFe-LDH/TFO samples. Figure 4a shows a linear relationship of (1/C)^2^ vs. the potential. Compared with α-Fe_2_O_3_, all the other photoanodes show distinctly smaller slopes, with the smallest observed for CoFe-LDH/TFO. The carrier densities were calculated based on the Mott–Schottky slopes, and the results are listed in Table S1. However, it should be noted that these data were primarily suitable for comparison due to the accurate capacitance calculation based on the grounds of the flat structure [35,36]. Among the four photoanodes, CoFe-LDH/TFO exhibits the highest carrier density. The introduction of Ti as dopants obviously increases the carrier densities of hematite, promoting the separation of photogenerated electron–hole pairs. Additionally, CoFe-LDH, as cocatalysts, enhances the carrier densities of hematite, contributing to band bending at the electrode–electrolyte interface and accelerating interfacial charge transfer [37,38,39]. 

To gain insights into the impact of Ti doping and CoFe-LDH deposition on the charge transfer process, the EIS measurements were conducted at a potential of 1.23 V_RHE_ under light irradiation (Figure 4b). An equivalent circuit was employed to fit the EIS data (see inset of Figure 4b), and the fitting values are listed in Table 1. The Nyquist plots for the four photoanodes show two semicircles, representing the charge trapping resistance within the bulk and the charge transfer resistance at the interface between the photoanode and the electrolyte. An enlarged impedance view of CoFe-LDH/TFO is shown in the inset of Figure 4b. Ti doping reduces the R_bulk_ from 391.9 Ω to 93.7 Ω, indicating that Ti dopants reduce the charge trapping resistance in the bulk [40]. CoFe-LDH plays a crucial role in hole transport into the electrolyte, evident from the significantly lower R_ct_ value of CoFe-LDH/α-Fe_2_O_3_ (283.7 Ω) compared to α-Fe_2_O_3_ (613.3 Ω) and TFO (475.6 Ω). Compared to the other three photoanodes, the synergistic effects of Ti doping and CoFe-LDH cocatalysts result in a remarkable decrease in the R_bulk_ and R_ct_ values of CoFe-LDH/TFO [36]. Notably, the ratios of the R_bulk_ and R_ct_ of Ti-doped CoFe-LDH/α-Fe_2_O_3_ to α-Fe_2_O_3_ are higher than those of non-Ti-doped CoFe-LDH/α-Fe_2_O_3_ to α-Fe_2_O_3_, suggesting that Ti doping enhances the electronic conductivity of α-Fe_2_O_3_ and may promote the surface charge transfer effect of the CoFe-LDH cocatalysts on the photoanodes.

In order to further confirm the improved charge transfer and separation of CoFe-LDH/TFO, the role of Ti doping in the interfacial charge separation and transfer process was investigated. The LSV measurements used 1 M Na_2_SO_3_ as a hole scavenger under light irradiation. The rapid hole injection kinetics during the oxidation of Na_2_SO_3_ allow for the complete capture of the separated holes [3,41], the charge separation efficiencies in the bulk (η_sep_) and the charge injection efficiencies on the surface (η_inj_) can be calculated using Equations (4)–(6), and the related plots are provided in Figure S3. As shown in Figure 4c,d, the η_sep_ values for the TFO and CoFe-LDH/TFO photoanodes at 1.23 V_RHE_ are 31.2% and 36.1%, respectively, which are significantly higher than the values of 17.4% for α-Fe_2_O_3_ and 25.5% for CoFe-LDH/α-Fe_2_O_3_. This suggests that Ti doping enhances the electron–hole separation in hematite, and the introduction of CoFe-LDH further improves this separation through hole extraction [42]. Regarding the charge injection on the surface, there is a substantial increase in the η_inj_ after CoFe-LDH deposition, from 36.2% to 63.2% at 1.23 V_RHE_. This increases CoFe-LDH’s ability to promote hole injection into the electrolyte for water oxidation. The η_inj_ of TFO also shows a slight increase compared to that of α-Fe_2_O_3_ at 1.23 V_RHE_. Notably, the difference in the charge injection efficiencies between CoFe-LDH/TFO and TFO is higher than that between CoFe-LDH/α-Fe_2_O_3_ and α-Fe_2_O_3_. This suggests that Ti doping significantly enhances the charge injection in CoFe-LDH/α-Fe_2_O_3_ compared with α-Fe_2_O_3_. In other words, Ti doping enhances the cocatalytic ability of CoFe-LDH for hematite photoanodes, consistent with the results from the EIS.

### 3.4. The Promoting Mechanism of CoFe-LDH/TFO Photoanodes for Water Oxidation

**Figure 5:**
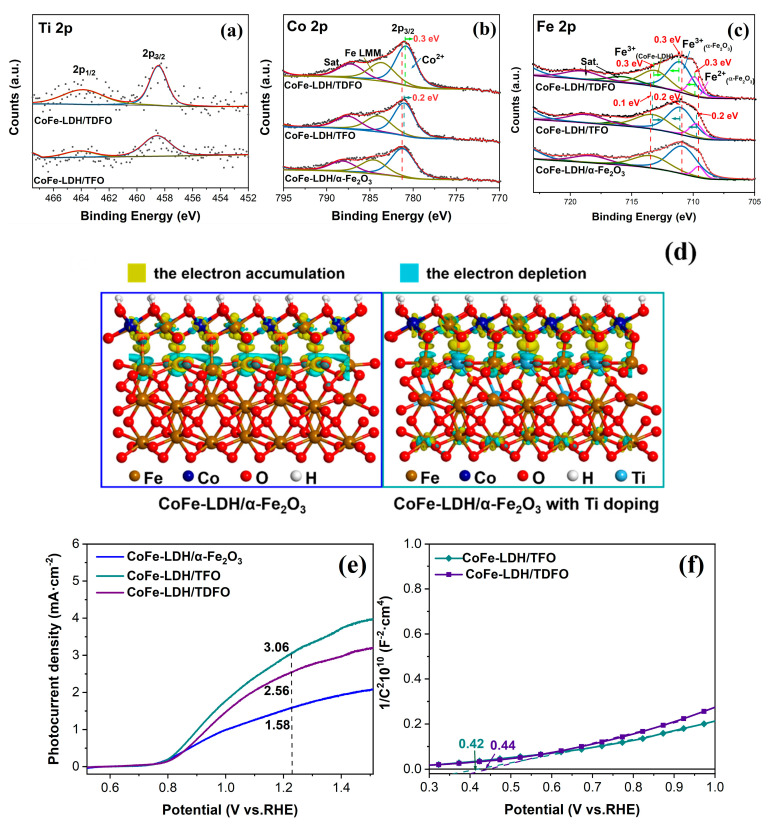
High-resolution XPS spectra of (**a**) Ti 2p of CoFe-LDH/TFO and CoFe-LDH/TDFO samples; (**b**) Co 2p and (**c**) Fe 2p of CoFe-LDH/α-Fe_2_O_3_, CoFe-LDH/TFO and CoFe-LDH/TDFO samples; (**d**) the charge density difference between the optimized structures of CoFe-LDH/α-Fe_2_O_3_ and CoFe-LDH/α-Fe_2_O_3_ with Ti doping; (**e**) LSV curves of CoFe-LDH/α-Fe_2_O_3_, CoFe-LDH/TFO and CoFe-LDH/TFO photoanodes; (**f**) Mott–Schottky plots for CoFe-LDH/TFO and CoFe-LDH/TFO photoanodes.

It has been discussed and proven that surface modification with CoFe-LDH enhances the charge separation and transfer efficiencies of TFO. The remarkably enhanced photocurrent density observed after CoFe-LDH deposition can be attributed to several factors. Firstly, the good optical transparency (Figure S1d) of CoFe-LDH reduces light absorption losses in the photoanodes, facilitating the formation of more photogenerated holes and electrons [19,43]. Additionally, we found the presence of an interfacial coupling between CoFe-LDH and α-Fe_2_O_3_. In order to study the effect of Ti doping on the CoFe-LDH/α-Fe_2_O_3_ photoanodes, we prepared CoFe-LDH/TFO with a double amount of Ti doping (denoted as CoFe-LDH/TDFO) using the same synthesis method as for CoFe-LDH/TFO (the Ti 2p XPS spectra of CoFe-LDH/TFO and CoFe-LDH/TDFO are exhibited in Figure 5a). The high-resolution Co 2p and Fe 2p XPS spectra of CoFe-LDH/α-Fe_2_O_3_ and CoFe-LDH/TDFO were investigated, as shown in Figure 5b,c, and the corresponding peak fitting results were similar to those of CoFe-LDH/TFO [29,30,34]. However, in the XPS spectra of Co 2p_3/2_ in Figure 5b, we observed noticeable negative shifts of 0.13 eV for CoFe-LDH/TFO and 0.28 eV for CoFe-LDH/TDFO compared to CoFe-LDH/α-Fe_2_O_3_. With increased Ti doping, these negative shifts became more pronounced. Furthermore, in Figure 5c, we observed that the Fe 2p_3/2_ peaks of α-Fe_2_O_3_ shifted towards higher binding energy with increasing Ti doping, while the Fe 2p_3/2_ peaks of CoFe-LDH shifted towards lower binding energy. These observations indicate the presence of the interface coupling effect between CoFe-LDH and α-Fe_2_O_3_ with Ti doping [44]. Notably, stronger coupling was observed with increased Ti doping, resulting in a partial electron transfer from α-Fe_2_O_3_ to CoFe-LDH, which explains the observed shifts [45].

The first-principles calculations were carried out to gain insights into the electron interactions between CoFe-LDH and α-Fe_2_O_3_. The optimized structures of CoFe-LDH/α-Fe_2_O_3_ with and without Ti doping are shown in Figure S4. To further investigate the effects of Ti doping, we examined the charge density differences over CoFe-LDH/α-Fe_2_O_3_ and CoFe-LDH/α-Fe_2_O_3_, as illustrated in Figure 5d. In both cases, we observed electron depletion regions (depicted in blue) and the electron accumulation regions (depicted in yellow). Notably, the yellow regions (electron accumulation) were mainly located on CoFe-LDH, while the blue regions (electron depletion) were mainly located on α-Fe_2_O_3_. This suggests that there is an electron transfer occurring between CoFe-LDH and α-Fe_2_O_3_, both with and without Ti doping [46].

Furthermore, the changes in charge density for CoFe-LDH and α-Fe_2_O_3_ can be observed in Figure 5d. After Ti doping, the number of electrons transferred between CoFe-LDH and α-Fe_2_O_3_ increased from 0.969 to 1.024, based on quantitative Bader charge analysis. This indicates a stronger electron transfer between CoFe-LDH and α-Fe_2_O_3_ after Ti doping. These results are consistent with the conclusions drawn from the XPS analysis (Figure 5b,c). In addition, the results are supported by the quantitative analysis of the calculated average Bader charge (Table S2). The valences of Co and Fe in Ti-doped CoFe-LDH decreased from 1.269 to 1.195 and from 1.503 to 1.378, respectively, indicating an increase in the number of valence electrons for Co and Fe. This is caused by the stronger electron shielding effect caused by Ti doping and aligns with the XPS results mentioned above [47].

Based on the results of the XPS analysis and the theoretical calculations, it was found that CoFe-LDH has interface coupling interactions with α-Fe_2_O_3_, which are further enhanced by Ti doping. The electron accumulation in CoFe-LDH facilitates the extraction of photo-induced holes and repels electrons. The stronger electron accumulation induced by Ti doping accelerates the charge transfer, allowing CoFe-LDH to accept holes more easily [46,48]. This explains the higher surface charge injection efficiency observed after Ti doping. However, despite the strongest electron coupling interaction between CoFe-LDH and α-Fe_2_O_3_ in CoFe-LDH/TDFO, it exhibited only a moderate photocurrent density (Figure 5e). The reason for the lower photocurrent density in CoFe-LDH/TDFO compared with CoFe-LDH/TFO can be attributed to the positive shift in the flat-band potential and the decrease in the carrier density, according to the Mott–Schottky plots in Figure 5f. These results indicate that Ti doping finely regulates the electronic structure at the interface between CoFe-LDH and α-Fe_2_O_3_, enhancing the interfacial charge separation and transfer. Consequently, this enhancement leads to the superior PEC performance of CoFe-LDH/TFO for water oxidation.

**Scheme 1:**
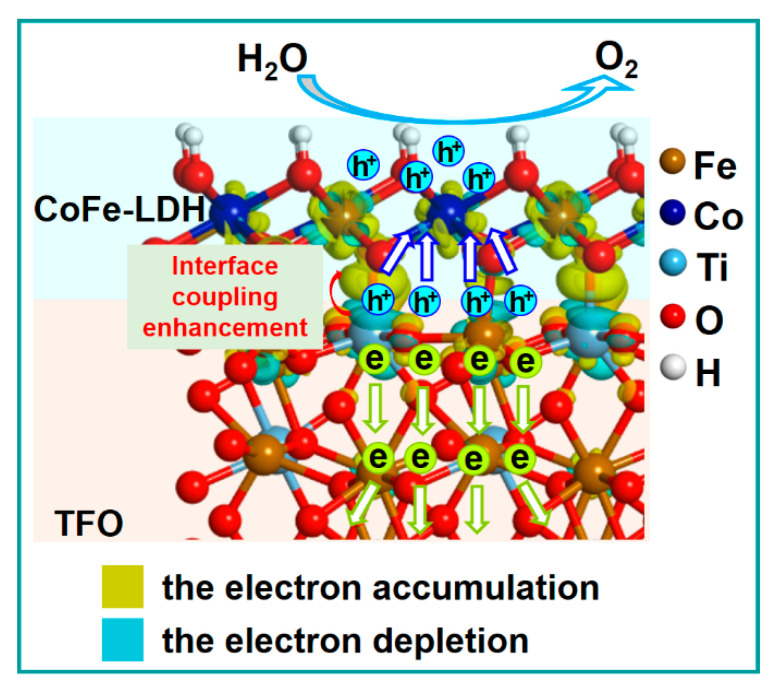
The charge transfer paths supposed to be occurring in CoFe-LDH/TFO photoanode for PEC water oxidation.

Based on the preceding discussions, we have illustrated the charge transfer pathways for CoFe-LDH/TFO photoanodes in Scheme 1 to clarify the enhancement of the photoelectrochemical water oxidation performance. Under light irradiation, α-Fe_2_O_3_ is excited, generating photoinduced electron–hole pairs. When Ti is doped into hematite, the increased electronic conductivity in the bulk enables the photogenerated electrons and holes to migrate more rapidly to the fluorine-doped tin oxide substrate and the surface. Subsequently, CoFe-LDH is deposited onto the hematite surface. Thanks to the enhanced electron accumulation within CoFe-LDH, which is a result of Ti doping, the photogenerated holes can be extracted more efficiently to CoFe-LDH, limiting carrier recombination [8,47]. Co ions can act as active sites for receiving holes from α-Fe_2_O_3_. These received holes are then oxidized to high-valent states [49]. Because of the presence of electron accumulation, Co sites become more receptive to accepting holes. This increased receptivity facilitates Co ions in delivering a positive charge, which, in return, promotes the formation of reactant OOH intermediates, thus accelerating O_2_ evolution [8,48].

## 4. Conclusions

In summary, we have demonstrated the important role of Ti-doped hematite photoanodes with CoFe-LDH deposited on the surface, which were successfully synthesized and applied to PEC water oxidation. The resulting CoFe-LDH/TFO photoanode exhibited an outstanding photocurrent density of 3.06 mA/cm^2^ at 1.23 V_RHE_, which is five times higher than that of pristine hematite. The charge separation of the bulk and the injection efficiencies of the surface were increased up to 36.1% and 80.1%, respectively, compared with those of hematite (17.4% and 36.2%). The enhancement of the PEC performance can be ascribed to the interface interaction between CoFe-LDH and Ti:α-Fe_2_O_3_. Ti doping plays a dual role: it improves the electronic conductivity of hematite and enhances the interface coupling effect, as well as regulating the interfacial electron structure between CoFe-LDH and α-Fe_2_O_3_. These combined effects effectively accelerate both the bulk and surface charge transfer processes.

## Figures and Tables

**Table 1:** The equivalent circuit model EIS results for different photoanodes.

Photoanodes	R_s_ (Ω)	R_bulk_ (Ω)	R_ct_ (Ω)
α-Fe_2_O_3_	30.5	391.9	613.3
TFO	29.8	93.7	475.6
CoFe-LDH/α-Fe_2_O_3_	27.5	228.8	283.7
CoFe-LDH/TFO	22.5	32.4	96.6

## Data Availability

Not applicable.

## Supplementary Materials

The following supporting information can be downloaded at: https://www.mdpi.com/article/10.3390/nano13182579/s1, Figure S1: (a) The cross-sectional SEM image of α-Fe_2_O_3_, (b) EDS elemental mapping in a selected region of CoFe-LDH/TFO, (c) XRD spectra of TFO, CoFe-LDH/TFO and CoFe-LDH/TDFO films on the fluorine-doped tin oxide glass, (d) DR UV–Vis spectra of α-Fe_2_O_3_, TFO, CoFe-LDH/α-Fe_2_O_3_ and CoFe-LDH/TFO. Figure S2: The photocurrent stability test for CoFe-LDH/TFO photoanode. Figure S3: (a) The LSV curves of α-Fe_2_O_3_, TFO, CoFe-LDH/α-Fe_2_O_3_ and CoFe-LDH/TFO photoanodes with Na_2_SO_3_ as the hole scavenger; (b) the light harvesting efficiency (LHE) curves of α-Fe_2_O_3_, TFO, CoFe-LDH/α-Fe_2_O_3_ and CoFe-LDH/TFO samples; (c) the energy density flux of AM 1.5 G solar; and (d) the calculated current density flux of (a) α-Fe_2_O_3_, (b) TFO, (c) CoFe-LDH/α-Fe_2_O_3_ and (d) CoFe-LDH/TFO. Figure S4: The optimized structures of CoFe-LDH/α-Fe_2_O_3_ and CoFe-LDH/α-Fe_2_O_3_ with Ti doping. Table S1: The calculated results on the carrier density and flat-band potential of α-Fe_2_O_3_, TFO, CoFe-LDH/α-Fe_2_O_3_ and CoFe-LDH/TFO from the Mott–Schottky measurements. Table S2: The calculated average Bader charge of the Co atom and Fe atom. Refs. [8,35,50,51,52,53,54] are cited in the Supplementary Materials.
