# Supplementary material for: Interface Engineering of CoFe-LDH Modified Ti: α-Fe2O3 Photoanode for Enhanced Photoelectrochemical Water Oxidation
Source: Nanomaterials (Basel). 2023 Sep 18;13(18):2579. doi: 10.3390/nano13182579 (PMC10536217; doi:10.3390/nano13182579)
Supplement: Supplementary file 1 [file nanomaterials-13-02579-s001.zip › nanomaterials-2581777-supplementary.pdf]

nanomaterials

Supplementary Materials

Interface Engineering of CoFe-LDH Modified Ti:α-Fe_2O_3 Photoanode for Enhanced Photoelectrochemical Water Oxidation

Yue Chang [1,2,3,*], Minmin Han [4,5], Yehui Ding [1], Huiyun Wei [6], Dawei Zhang [1,2,3], Hong Luo [1,2], Xiaogang Li [1,2] and Xiongbo Yan [7]

[1] Institute of Advanced Materials and Technology, University of Science and Technology Beijing, Beijing 100083, China; dingsdfzustb@outlook.com (Y.D.); dzhang@ustb.edu.cn (D.Z.); luohong@ustb.edu.cn (H.L.); lixiaogang99@263.net (X.L.)
[2] National Materials Corrosion and Protection Data Center, University of Science and Technology Beijing, Beijing 100083, China
[3] BRI Southeast Asia Network for Corrosion and Protection (MOE), Shunde Innovation School, University of Science and Technology Beijing, Foshan 528399, China
[4] National Engineering Research Center for Intelligent Electrical Vehicle Power System, College of Mechanical and Electrical Engineering, Qingdao University, Qingdao 266071, China; han90v@163.com
[5] International Center for Materials Nanoarchitectonics (WPI-MANA), National Institute for Materials Science (NIMS), 1-1 Namiki, Tsukuba 305-0044, Ibaraki, Japan
[6] School of Mathematics and Physics, University of Science and Technology Beijing, Beijing 100083, China; huiyunwei@ustb.edu.cn
[7] Beijing Advanced Innovation Center Materials Genome Engineering, University of Science and Technology Beijing, Beijing 100083, China; yanxiongbo@ustb.edu.cn
* Correspondence: changyue@ustb.edu.cn

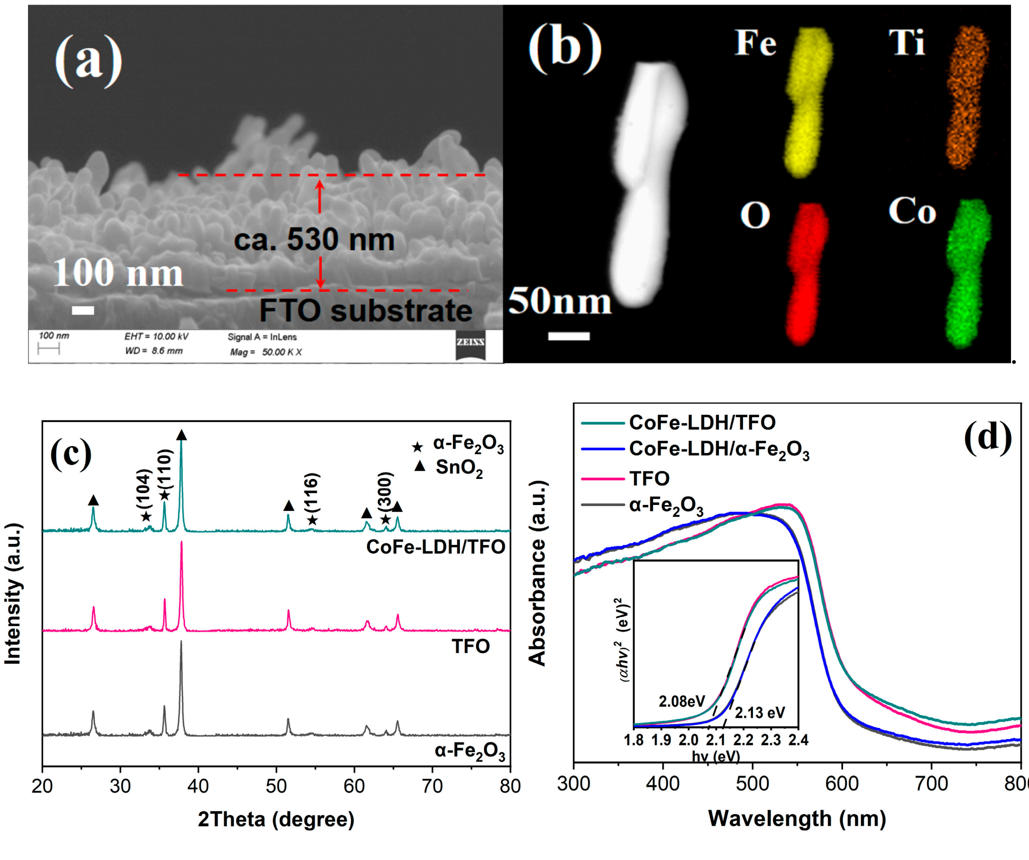

Figure S1. **(a)** The cross-sectional SEM image of α-Fe_2O_3, **(b)** EDS elemental mapping in a selected region of CoFe-LDH/TFO. **(c)** XRD spectra of TFO, CoFe-LDH/TFO and CoFe-LDH/TDFO films on

the fluorine-doped tin oxide glass; **(d)** DR UV-Vis spectra of α-Fe_2O_3, TFO, CoFe-LDH/α-Fe_2O_3 and CoFe-LDH/TFO.

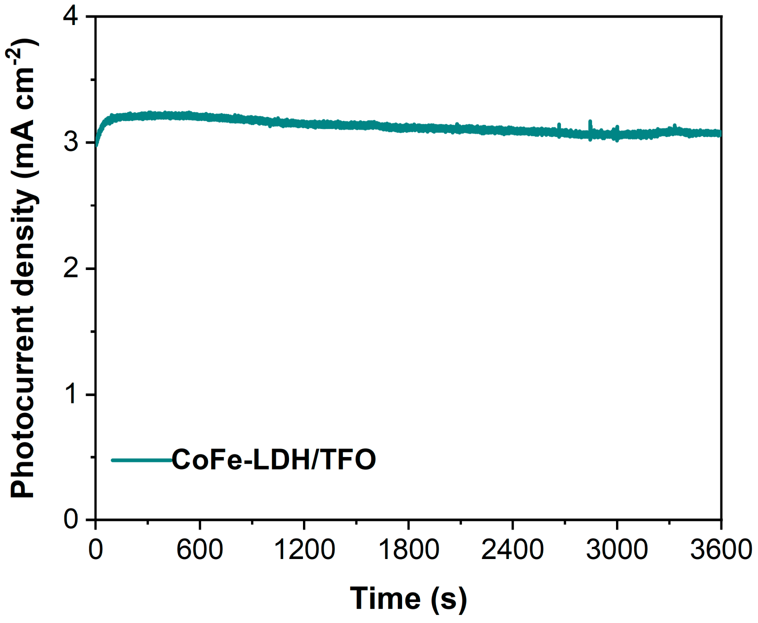

Figure S2. The photocurrent stability test for CoFe-LDH/TFO photoanode.

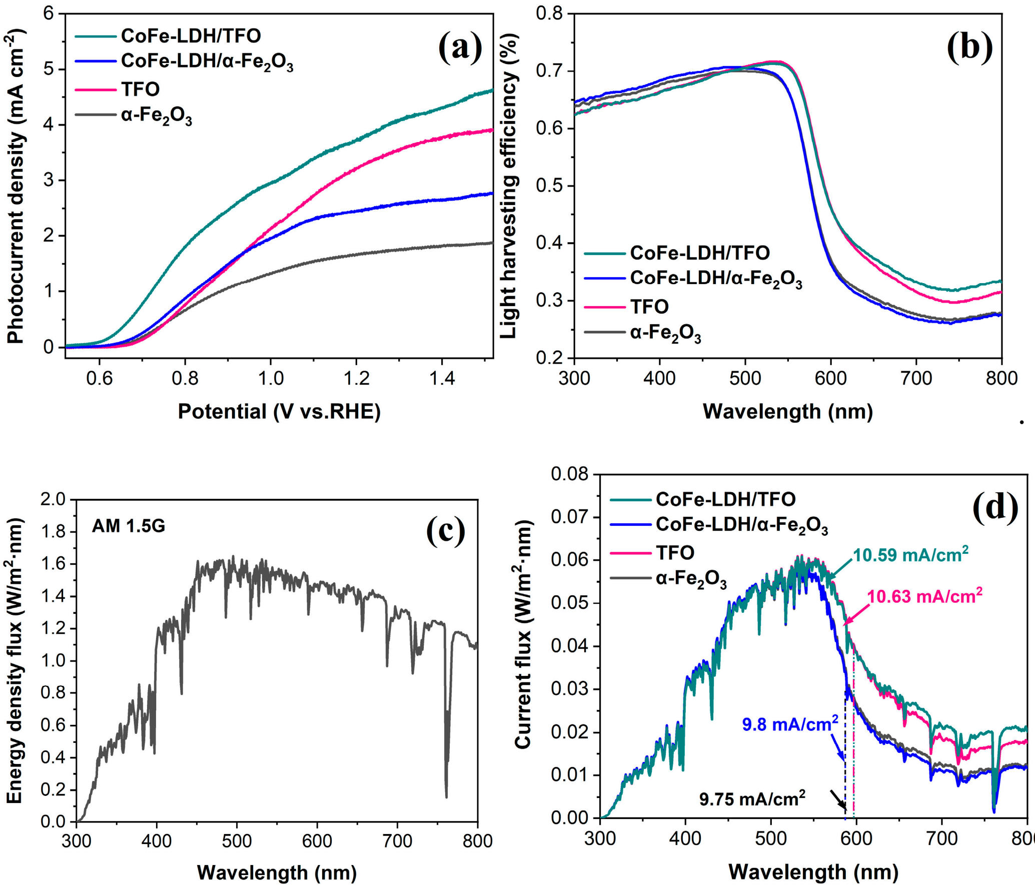

Figure S3. (a) LSV curves of of α-Fe_2O_3, TFO, CoFe-LDH/α-Fe_2O_3 and CoFe-LDH/TFO photoanodes with Na_2SO_3 as the hole savenger; **(b)** Light harvesting efficiency (LHE) curves of α-Fe_2O_3, TFO, CoFe-LDH/α-Fe_2O_3 and CoFe-LDH/TFO samples; **(c)** the energy density flux of AM 1.5 G solar and

(d) the calculated current density flux of (a) α-Fe_2O_3, (b) TFO, (c) CoFe-LDH/α-Fe_2O_3 and (d) CoFe-LDH/TFO.

J_{abs} was calculated according to the overlapped area between UV-Vis absorption spectrum and AM 1.5 G solar spectrum by the following Equation (S1) and (S2) [1,2]:

$$J_{abs} = \int \frac{\lambda}{1240} \times \varphi AM1.5G(\lambda) \times LHEd\lambda \quad (1)$$

$$LHE = 1 - 10^{-A(\lambda)} \quad (2)$$

where λ represents the wavelength (nm), $\varphi AM1.5G(\lambda)$ provides the simulated solar spectral irradiance (W/(m^2·nm)), LHE is the light harvesting efficiency, and $A(\lambda)$ is the absorbance at wavelength λ.

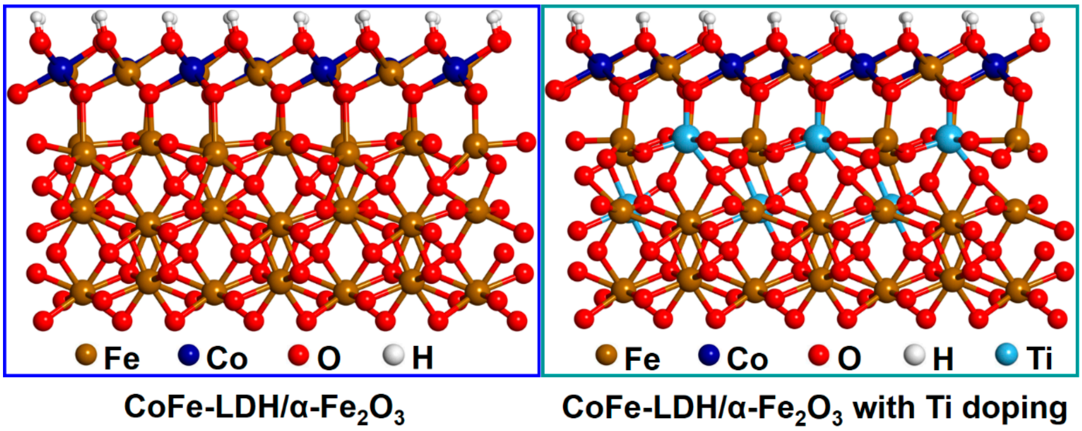

Figure S4. The optimized structures of CoFe-LDH/α-Fe_2O_3 and CoFe-LDH/α-Fe_2O_3 with Ti doping.

Calculation Method

First-principle calculations were performed by the density functional theory (DFT) using the Vienna Ab-initio Simulation Package (VASP) package [3]. The generalized gradient approximation (GGA) with the Perdew-Burke-Ernzerhof (PBE) functional were used to describe the electronic exchange and correlation effects [4-6]. Uniform G-centered k-points meshes with a resolution of $2\pi \times 0.05$ Å^{-1} and Methfessel-Paxton electronic smearing were adopted for the integration in the Brillouin zone for geometric optimization. The simulation was run with a cutoff energy of 500 eV throughout the computations. These settings ensure convergence of the total energies to within 1 meV per atom. Structure relaxation proceeded until all forces on atoms were less than 10 meV Å^{-1} and the total stress tensor was within 0.03 GPa of the target value. In order to study the phenomenon of Co peak shift towards low binding energy in XPS, we constructed Fe_2O_3/CoFe-LDH heterostructure, in which Fe_2O_3 selects (104) crystal plane and CoFe-LDH selects (001) crystal plane.

Table S1. The calculated results of the carrier density and flat band potential of α-Fe_2O_3, TFO, CoFe-LDH/α-Fe_2O_3 and CoFe-LDH/TFO by the mott-schottky measurements.

Photoanodes	Carrier density (10^{19} cm^{-3})
**α-Fe_2O_3**	2.99
**TFO**	20.4
**CoFe-LDH/α-Fe_2O_3**	9.93
**CoFe-LDH/TFO**	50.9
**CoFe-LDH/TDFO**	39.1

The carrier density (N_d) can be calculated according to Mott-Schottky plots using the following equation (3) [1,7]:

$$N_d = \frac{2/e_0\varepsilon\varepsilon_0}{d(1/C^2)/dV} \quad (3)$$

where e_0 is the electronic charge constant (1.6 × 10^{-19} C), ε is the relative permittivity constant (80 for α-Fe_2O_3), ε_0 is the vacuum permittivity constant (8.86 × 10^{-14} F/cm).

Table S2. The calculated averaged Bader charge of Co atom and Fe atom.

**Atom**	**Charge (e)**	
	**Without Ti doping**	**With Ti doping**
**Co in CoFe-LDH**	+1.269	+1.195
**Fe in CoFe-LDH**	+1.503	+1.378
**Fe in α-Fe_2O_3**	+1.428	+1.438

References:

1. Yang, G.L.; Li, Y.X.; Pang, H.; Chang, K.; Ye, J.H. Ultrathin Cobalt–Manganese Nanosheets: An Efficient Platform for Enhanced Photoelectrochemical Water Oxidation with Electron-Donating Effect. *Adv. Funct. Mater.* **2019**, *29*, 1904622.
2. Zhang, K.; Dong, T.; Xie, G.; Guan, L.; Guo, B.; Xiang, Q.; Dai, Y.; Tian, L.; Batool, A.; Jan, S.U.; Boddula, R.; Thebo, A.A.; Gong, J.R. Sacrificial Interlayer for Promoting Charge Transport in Hematite Photoanode. *ACS Appl. Mater. Inter.* **2017**, *9*, 42723–42733.
3. Kresse, G.; Furthmüller, J. Efficiency of Ab-Initio Total Energy Calculations for Metals and Semiconductors Using a Plane-Wave Basis Set. *Comput. Mater. Sci.* **1996**, *6*, 15–50.
4. Perdew, J.P.; Burke, K.; Ernzerhof, M. Generalized gradient approximation made simple. *Phys. Rev. Lett.* **1996**, *77*, 3865–3868.
5. Blöchl, P.E. Projector augmented-wave method. *Phys. Rev. B Condens. Matter Mater. Phys.* **1994**, *50*, 17953–17979.
6. Kresse, G.; Joubert, D. From Ultrasoft Pseudopotentials to the Projector Augmented-Wave Method. *Phys. Rev. B Condens. Matter Mater. Phys.* **1999**, *59*, 1758.
7. Ravishankar, S.; Bisquert, J.; Kirchartz, T. Interpretation of Mott–Schottky plots of photoanodes for water splitting. *Chem. Sci.* **2022**, *13*, 4828–4837.
